# Use of *De Novo* Transcriptome Libraries to Characterize a Novel Oleaginous Marine *Chlorella* Species during the Accumulation of Triacylglycerols

**DOI:** 10.1371/journal.pone.0147527

**Published:** 2016-02-03

**Authors:** Cresten B. Mansfeldt, Lubna V. Richter, Beth A. Ahner, William P. Cochlan, Ruth E. Richardson

**Affiliations:** 1 Department of Biological and Environmental Engineering, Cornell University, Ithaca, NY, United States of America; 2 Romberg Tiburon Center for Environmental Studies, San Francisco State University, Tiburon, CA, United States of America; 3 Department of Civil and Environmental Engineering, Cornell University, Ithaca, NY, United States of America; Chinese Academy of Medical Sciences, Peking Union Medical College, CHINA

## Abstract

Marine chlorophytes of the genus *Chlorella* are unicellular algae capable of accumulating a high proportion of cellular lipids that can be used for biodiesel production. In this study, we examined the broad physiological capabilities of a subtropical strain (C596) of *Chlorella* sp. “SAG-211-18” including its heterotrophic growth and tolerance to low salt. We found that the alga replicates more slowly at diluted salt concentrations and can grow on a wide range of carbon substrates in the dark. We then sequenced the RNA of *Chlorella* strain C596 to elucidate key metabolic genes and investigate the transcriptomic response of the organism when transitioning from a nutrient-replete to a nutrient-deficient condition when neutral lipids accumulate. Specific transcripts encoding for enzymes involved in both starch and lipid biosynthesis, among others, were up-regulated as the cultures transitioned into a lipid-accumulating state whereas photosynthesis-related genes were down-regulated. Transcripts encoding for two of the up-regulated enzymes—a galactoglycerolipid lipase and a diacylglyceride acyltransferase—were also monitored by reverse transcription quantitative polymerase chain reaction assays. The results of these assays confirmed the transcriptome-sequencing data. The present transcriptomic study will assist in the greater understanding, more effective application, and efficient design of *Chlorella*-based biofuel production systems.

## Introduction

As the market for biofuels expands, photosynthetic microalgae can be potentially employed in third-generation biofuel production at commercial scales [1]. Single-celled algae have received heightened attention because of their fast growth rates, high photosynthetic efficiencies, and rapid lipid accumulation [2, 3]. Many algal species are capable of biosynthesizing and storing neutral lipids composed primarily of triacylglycerides (TAGs) [4–6], which can be extracted and industrially transesterified into biodiesel [7]. Algae bioaccumulate TAGs as a means to store excess energy when cells experience a non-carbon nutrient limitation that prevents cell doubling (i.e., phosphate or nitrate limitation) [8, 9]. Therefore, the accumulation of TAGs is decoupled from the exponential growth of the organism, and genes encoding enzymes contributing to TAG accumulation are tightly controlled [10–13].

The accumulation of lipids by individual species of chlorophytes or green algae under environmentally stressful conditions is well established in the literature (e.g., [14, 15]). Within the green algae, for which we have a well-established genetically tractable model system (*Chlamydomonas reinhardtii*), representatives of the *Chlorella* genus have been shown to bioaccumulate TAGs efficiently. *Chlorella* strains have therefore been investigated for potential biodiesel production at commercial scales [16, 17], and several industrial *Chlorella* applications for biofuel production have been reported [18, 19]. However, limited genomic information is available for the *Chlorella* genus. Three full genomes for *Chlorella* are currently available in the NCBI database: *Chlorella variabilis* NC64A [20], a photobiont initially sequenced to study viral/algal interactions, *Chlorella vulgaris*, and *Chlorella pyrenoidosa* [21]. One additional strain in a related genus, *Auxenochlorella protothecoides*, was recently sequenced [22]. Additional *Chlorella* sequencing projects of various levels of completeness have been deposited in other databases (such as greenhouse.lanl.gov/). To yield information about the physiology of other *Chlorella* species without *a priori* knowledge of the genome, advanced sequencing technologies (e.g., Illumina RNA-seq) and sequence assembly methods allow high-throughput *de novo* transcriptomic analyses.

Most previous transcriptomic studies of various *Chlorella* species utilized targeted transcriptomic approaches such as reverse-transcription quantitative PCR (RT-qPCR) to monitor pre-selected targets [23–25]. However, analyses of the full transcriptome using next-generation sequencing technologies are more useful because they can provide a global overview of the response of *Chlorella* cultures to various environmental states, and potentially link the expression of specific and large sets of transcripts with phenotypic responses. This approach is an emerging and widely applied tool that has been used to investigate the differences between autotrophic and heterotrophic growth in *A. protothecoides* [22], the growth of *C. pyrenoidosa* under salt stress [26], the transition from starch to lipid synthesis in *C. pyrenoidosa* [21], the growth of *Chlorella sorokiniana* under high carbon dioxide concentrations [27], and to survey the transcriptome of *Chlorella minutissima* UTEX2341 in general [28].

In the current study, we have focused on a new *Chlorella* strain (C596) obtained from the University of Hawaii and selected from among hundreds of isolates as a promising candidate for biodiesel production (Johnson et al., in review [29]). This study had three main goals: (1) to investigate the growth characteristics of C596, (2) to elucidate key metabolic pathway genes including the genes responsible for lipid biosynthesis, and (3) to discover specific genes that are involved in TAG accumulation. To better understand the growth characteristics of this marine algae, strain C596 was grown using media of various salt concentrations (35, 17, 8.8, and 4.4 parts per thousand (ppt)) and media enriched with organic substrates (acetate, glycerol, glucose, sucrose, or succinate) in the dark. To elucidate genes involved in the biosynthesis of lipids, strain C596 was subjected to a co-limitation of nitrogen and phosphorus to promote subsequent TAG accumulation, and the transcriptome expressed by the culture was monitored during a sequential co-limitation of phosphorus and nitrogen using Illumina RNA-seq technology. To determine specific genes involved in TAG accumulation, comparisons between RNA pools highlighted differentially expressed genes responding to nutrient stresses, and follow-up RT-qPCR studies confirmed several of these findings.

## Materials and Methods

### *Chlorella* strain C596 Strain Information

*Chlorella* sp. “SAG 211–18” [30] strain C596 was obtained from the University of Hawaii culture collection. This strain was recovered from Portlock Point, O’ahu, Hawaii by S. Brown in 2009 and identified by C. J. O’Kelly (personal communication). Cultures were initially purified by single cell-sorting, and then grown in 25-mL sterile, polystyrene, tissue culture flasks with vented caps (Falcon 353107; Corning Inc., Corning, NY, USA) at 19.0 ± 0.5°C within a temperature-controlled environmental chamber on a 12:12 h light:dark cycle of approximately 50 μmol photons m^−2^ s^−1^. Cultures were grown using coastal seawater collected from Half Moon Bay, CA, USA. The seawater was previously filtered (0.2-μm filters; Whatman PolyCap 150 TC; GE Healthcare Bio-Sciences, Pittsburgh, PA, USA), and enriched according to *f*/2 medium [31, 32] as previously outlined [33], but with greater concentrations of both phosphate (86.4 μmol L^−1^) and silicate (1,305 μmol L^−1^).

### Nomenclature

The electronic version of this article in Portable Document Format (PDF) in a work with an ISSN or ISBN represents a published work according to the International Code of Nomenclature for algae, fungi, and plants, and hence the new names contained in the electronic publication of a PLOS ONE article are effectively published under that Code from the electronic edition alone, so there is no longer any need to provide printed copies. The online version of this work is archived and available from the following digital repositories: PubMed Central and LOCKSS.

### C596 Culturing Conditions

At Cornell University, *Chlorella* cells for experimentation were routinely cultured in Aquil (synthetic seawater) medium with a salinity of 35 ppt containing 300 μmol L^−1^ nitrate, 10 μmol L^−1^ phosphate, and prepared according to Price et al. [34]. *Chlorella* strain C596 cultures were incubated at 25°C open to the atmosphere and under a constant illumination of 80 μmol photons m^−2^ s^−1^. Synthetic seawater was diluted with Milli-Q ultra-pure water (EMD Millipore Corporation, Billerica, MA, USA) to test the salinity tolerance of strain C596 at reduced salinity levels (17, 8.8, and 4.4 ppt). When testing for heterotrophic growth, either glucose, succinate (as sodium succinate), sucrose, glycerol, or acetate (as sodium acetate) was filter-sterilized and added to the sterile Aquil media at a final concentration of 20 mmol L^−1^, and the cultures were incubated in the dark at 25°C.

For the nutrient-limitation experiments performed at San Francisco State University, batch cultures of strain C596 were grown in 6-L glass (Pyrex) flat-bottomed boiler flasks (Fisher Science, Pittsburgh, PA, USA) containing 5-L of basal medium. The basal medium consisted of sterile-filtered (0.2-μm Whatman PolyCap 150 TC) natural seawater (collected from Bodega Bay, CA) and enriched with ESNW medium [35–37] as previously outlined [33] with the following modifications: 39.3 nmol L^−1^ copper added as CuSO_4_⋅5H_2_O; 64.0 nmol L^−1^ phosphorus added as KH_2_PO_4_; 13.1 μmol L^−1^ iron added as FeCl_3_⋅6H_2_O; and silicon was not added. Selenium (Na_2_SeO_3_) was added at 6.36 nmol L^−1^. Nitrogen was added as nitrate (NaNO_3_) to a final concentration of 814 μmol L^−1^. *Chlorella* cultures were grown within Versatile Environmental Test Chambers (Sanyo MLR-351H; Panasonic MLR-352H) under a programmable diel cycle (14:10 h L:D), with daytime and nighttime temperatures of 30 and 25°C, respectively. The temperature and photosynthetic photon flux density (PPFD) were gradually increased or decreased over 60 min to simulate natural sunrise and sunset conditions. The PPFD at the surface of the flasks was 800–820 μmol photons m^−2^ s^−1^ during the daytime, as measured with a 4-*π* collector (QSL-100 Quantum scalar irradiance meter; Biospherical Instruments Inc., San Diego, CA, USA). Cultures were stirred (60 rpm) using magnetic stir bars, and the pH of the cultures was maintained at or below 8.1 ± 0.1 by the direct injection of compressed CO_2_/air mixture (15% v/v; Praxair Gas, Danbury, CT, USA) into the media through a Pyrex course-fritted, glass dispersion tube (part 39533–12EC; Corning Inc., Corning, NY, USA).

Aseptic techniques were used throughout the culturing process for the batch and nutrient limitation experiments to minimize fungal growth and bacterial contamination. Culture growth was monitored through cell counts using a Neubauer hemacytometer (Spencer Bright Line) at 100× magnification with an inverted microscope (model IX83; Olympus Corp., Tokyo, Japan) and at 40× magnification with an optical microscope (model 1864; Southern Precision Instruments, San Antonio, TX, USA) for the nutrient limitation and batch experiments, respectively. These cell counts were combined with chloroplast fluorescence measurements using a 10-AU Fluorometer (Turner Designs, Sunnyvale, CA, USA). All cultures were performed in triplicate.

#### Sample Schedule

Cultures were sampled twice daily for dissolved nutrients and growth throughout the nutrient-replete exponential and the nutrient-limited stationary phase of the nutrient limitation experiments, whereas samples for lipids were only collected after ambient nutrient concentrations declined to levels considered limiting to growth. Samples for molecular analyses were taken during three phases of cell growth: the N-replete exponential phase on day four (86 h), the late exponential phase (transition to stationary growth phase induced by low N concentrations) on day five (109 h), and the N-stressed stationary phase on day eight (182 h), in which the cells had been depleted of external N for < 3 days. To ensure that stationary growth was induced by N and not P limitation, the N:P ratio of the media was adjusted to approximately 13:1 based on previously determined nutrient drawdown rates for strain C596 under these light and temperature conditions (data not shown).

#### Nutrient Analysis

Samples for nutrient analyses were filtered through combusted (450°C for 4.5 h) Whatman GF/F filters (Maidstone, UK). Nitrate plus nitrite (NO_3_^−^ + NO_2_^−^) and orthophosphate (PO_4_^3−^) concentrations were analyzed using a Lachat Instruments automated ion analyzer (8000 series; Hach Co., Loveland, CO, USA) with a standard colorimetric technique [38, 39].

#### Lipid Analysis

Samples for lipid and TAG analyses were collected by filtration onto combusted 47-mm glass fiber filters (691; VWR North American Cat No. 28333–129), gently rinsed thrice with 0.5 mol L^−1^ ammonium formate to remove external salts, and stored frozen at -80°C prior to express shipping on dry ice to the University of Hawaii (Dr. R. Bidigare). Here, the samples were analyzed using a microscale analytical approach for lipids and high performance liquid chromatography for TAGs; detailed procedures are outlined by Johnson et al. (in review) [29].

### Microscopy

Nile red stain was dissolved in DMSO (Sigma Aldrich, St. Louis, MO) at 1 mg mL^−1^ and stored in aliquots at -20°C in the dark. A 3 μmol mL^−1^ Nile red solution for staining was prepared by diluting and mixing 1 μL of the 1 mg mL^−1^ stock into 1-mL HHBS buffer (20 mmol L^−1^ HEPES in Hanks solution, pH 7). C596 culture volumes of 5–15 mL were sampled at different growth phases and centrifuged at 3000 × *g* for 15 min. The pelleted cells were stained with 500 μL of Nile red staining solution and left overnight in the dark at room temperature. Cells were washed four times for 15 minutes each with 500 μL of HHBS buffer under gentle shaking. Stained cells were observed under an Olympus BX41 fluorescence microscope (Hitschfel Instruments Inc., St Louis, MO) equipped with an Hg lamp, a Q-Imaging QICAM digital camera (Quantitative Imaging Corporation, Surrey, Canada), and image acquisition software (QCapture suite V2.60; Quantitative Imaging Corporation) using a Cy3 filter set and a 100× objective lens.

### RNA Extraction

In total, 1 mg (dry weight) of strain C596 cells were harvested by either filtration onto 0.2-μm pore size membrane filters (Nuclepore Track-Etch membrane, 47-mm diam. Whatman; GE Healthcare Bio-Sciences) at low vacuum pressure differential (< 5 psi), or by centrifugation at 4,000 × *g* for 30 min. Cells collected by filtration or centrifugation were re-suspended in seawater medium and re-centrifuged for 10 min at 11,600 × *g*. The resulting pellet was stored in 200 μL of RNA*later* (Ambion Life Technology, NY, USA) within RNase-free screw-cap microcentrifuge tubes at -20°C. Total RNA was extracted and purified using the RNeasy Plant Mini Kit (Qiagen, CA, USA) with the following modifications for cell lysis. Cell suspensions in RNA*later* were microcentrifuged for one minute at 20,000 × *g*, and the pellets were re-suspended in the kit-provided buffer RLT. Cells were lysed by agitation in a bead-beater (Mini-BeadBeater 16; Biospec Products, Bartlesville, OK) for four cycles of 40 s each using 0.5-mm glass beads that had been previously baked for four hours at 400°C [40]. The residual genomic DNA was removed using an on-column DNase digestion according to the manufacturer’s instruction (RNase-Free DNase Kit; Qiagen). The integrity and concentration of the total RNA was determined using a RNA 6000 Nano Kit on an Agilent Bioanalyzer 2100 (Agilent Technologies, Carlsbad, CA, USA). rRNA depletion was performed using the GeneRead rRNA Depletion Kit (Qiagen) for both bacterial and eukaryotic ribosomal RNA following the manufacturer’s instructions. A rRNA depletion kit was utilized instead of the dT-oligo bead method to better capture non-polyadenylated mRNA transcripts such as those from the chloroplast and mitochondrial genomes.

### 18S rRNA Sequencing and Phylogenetic Clustering

The 18S rRNA sequence for strain C596 was amplified using a set of universal eukaryotic primers [41] (S1 Table). The thermal cycling protocol was designed following the manufacturer’s instructions for the high-fidelity Phusion polymerase (New England Biolabs, Ipswich, MA, USA). The resulting amplicon was sequenced using the Sanger method at the Cornell University Core Genomics Facility. The phylogenetic relationship of the resulting 1800 bp 18S rRNA sequence for *Chlorella* strain C596 is determined through a distance tree created in MEGA 6 (http://www.megasoftware.net/) using Muscle. The sequences that strain C596 were compared against are included in S1 File.

### RNA Sequencing

The RNA samples were submitted to the Cornell University Core Genomics Facility for sequencing. After fragmentation and size selection following standard Illumina protocols, the fragments were sequenced using 250 base-pair (bp) paired-end reads on an Illumina MiSeq (Illumina, Inc., San Diego, CA, USA). The work-flow employed the T4 ligase method (TruSeq) on random hexamer amplified RNA.

### RNA Data Quality Analysis and Processing

The raw reads resulting from the RNA sequencing were first assessed for quality using FastQC (version 0.11.2, http://www.bioinformatics.babraham.ac.uk/projects/fastqc/). The files were then processed using Trimmomatic (version 0.30, http://www.usadellab.org/cms/?page = trimmomatic) to remove Illumina adapters and low-quality reads [42]. Low quality bps at the head of the sequences were trimmed. The data quality was checked using FastQC to confirm that the phred score is suitable for assembly and downstream analysis. All raw and processed datafiles considered in this study have been uploaded to NCBI’s Sequence Read Archive project SRP064130 under the BioProject ID PRJNA294811.

### RNA Transcript Assembly

The RNA reads were first screened for and filtered of rRNA using the program SortMeRNA (v 2.0, [43]). This analysis was used to bin the individual reads as originating from rRNA or other RNA. Approximately 60%-89% of reads for each sample were identified as rRNA even after employing an rRNA depletion kit. These rRNA reads were removed from the database, and the remaining reads were assembled into putative transcripts using the *de novo* assembly program Trinity (v 20140413p1; [44]). Trinity was run with the Jaccard_clip to reduce potential fusion genes. Multiple Trinity assemblies were run and each was analyzed for the presence of chimeras (by investigating the assembly of common housekeeping transcripts using the IGV Viewer [45]) and evaluated on several metrics including average length of sequences. The best Trinity assembly was generated using fifteen RNA pools from replicates (n = 2 to 3) of experiments (in total 11.4 million paired end reads yielding approximately 5.7 Gbp of sequence after trimming and filtering; excluding BioSamples SAMN04033435—SAMN04033437). This assembly was used for subsequent analyses (see S1 Fig for details) and resulted in a transcript library consisting of 131,470 predicted unique transcript fragments with an average length of 427 bp. Of these 131,470 transcripts, 13,036 (approximately 10%; S1 File) were assigned to our detected transcriptome in the overall BioProject dataset (at least five reads affiliated with a transcript in at least one sample). Combined, the detected transcriptome comprised 19.4 Mbp of sequence. The nutrient depletion time-course samples (BioSamples SAMN04033435—SAMN04033437; 4.1 million read pairs, 2.1 Gbp of sequence) were aligned to this transcriptome library.

### RNA Annotation

The resulting detected transcriptome (13,036; ≥ 5 reads per transcript) was annotated by several rounds of sequence homology analysis. The transcripts were first analyzed by the less computationally intense BLASTn search against the NCBI nt (nucleotide) database to identify and then remove potential bacterial sequences and known contaminants (e.g., human, mouse, and *Propinionbacterium acnes* sequences) prior to annotation when 85% identity or greater was displayed across the length of the transcript to a non-chlorophyta organism. The remaining transcripts were then compared against *A. protothecoides*, *C. variabilis* NC64A, and *C. reinhardtii* specific protein databases (7,198, 9,791, and 14,489 sequences, respectively) using the BLASTx algorithm available through NCBI (e-value cutoff was < 10^−10^). Additionally, to broaden the database for comparison, the transcripts were then BLASTx searched against the NCBI nr database; an e-value less than 10^−5^ was established as the cutoff for homolog consideration [26]. This final annotation was assigned using the Blast2GO program [46]. Only those transcripts identifed as homologus to a sequence from a Chlorophyta species were considered in the final expressed transcriptome (8,903 transcripts of the total 13,036 detected).

### RNA Transcript Analyses

Reads from the time-course samples considered in this study (BioSamples SAMN04033435—SAMN04033437) were mapped to the Trinity assembled library using the RSEM software package [47]. A transcript was considered expressed in a sample when the number of reads matching a transcript in the RSEM analysis (the expected value) exceeded four reads (≥ 5). Of the 8,903 transcripts that were identified as homologous to Chlorophyta, 6,554 transcripts exceeded this cutoff for the time-course samples.

The expected counts for transcripts that matched a Chlorophyta homolog were analyzed using edgeR [48] in the R software suite (v 3.1.2) to determine transcripts that were differentially regulated between samples. The edgeR analysis utilized a global normalization of the RNA reads by considering the trimmed mean of M-values between each pair of samples [48]. A transcript was considered differentially expressed when it displayed a fold-ratio greater than 2.0 or less than 0.5 with a p-value below 0.05, similar to cutoffs established previously [49]. The differentially expressed genes identified in the edgeR analysis were then assigned to KEGG (Kyoto Encyclopedia of Genes and Genomes) [50] categories using a tblastn search against the protein database for *C. variabilis*. The annotated KEGG categories for *C. variabilis* are freely available from www.genome.jp/kegg/. An e-value of 10^−10^ was established as the cutoff for this search.

Transcripts per million reads (TPM) values were used to make direct comparisons between samples. TPM values were normalized to the TPM value of the housekeeping gene encoding for cyclophillin and actin [51].

### RT-qPCR

The differential expressions of the galactoglycerolipid lipase and the diacylglyceride acyltransferase (DAGAT) genes were quantified via real-time quantitative PCR. Potentially contaminating DNA in the RNA samples was digested using a RNase-Free DNase Kit (Qiagen). Complimentary DNA (cDNA) was generated using an iScript reverse transcriptase and a blend of olig(dt) and random hexamer primers (iScript cDNA Synthesis Kit; Bio-Rad, Hercules, CA, USA). The DNase-treated RNA and the generated cDNA were assayed with PCR reactions to confirm the completion of the DNase digestion and the success of the reverse transcription, respectively. Calibration standard DNA samples were generated by PCR amplification using the cDNA as a template and primers specific to DAGAT, lipase, and the house-keeping gene cyclophillin. Primers were designed from assembled transcript sequences using Integrated DNA Technologies (IDT, Coralville, IA, USA), and the primer sequences are listed in S1 Table. The concentrations of the calibration standards were quantified using the PicoGreen assay (Quant-It PicoGreen dsDNA Reagent and Kit; Invitrogen, Grand Island, NY, USA). A series of dilutions, 10^2^ to 10^8^ copy number μL^−1^, were generated for each of the three DNA amplicons and were used for sample quantification. Triplicates of each of the calibration standard samples (for the genes of interest and cyclophillin) and the tested cDNA samples, in addition to duplicates of the negative controls (RNA and nuclease free water), were assayed for RT-qPCR on one 96 well plate. All RT-qPCRs were performed using the SsoAdvanced SYBR Green Supermix (Bio-Rad) on a BioRad CFx96 C100 machine. The log-linear equations of the calibration standard curves were used to quantify the copy number μL^−1^ of the target genes. DAGAT and lipase concentrations were ultimately normalized to the cyclopillin concentration measured for each tested condition.

## Results and Discussion

### Phylogenetics

**Fig 1:**
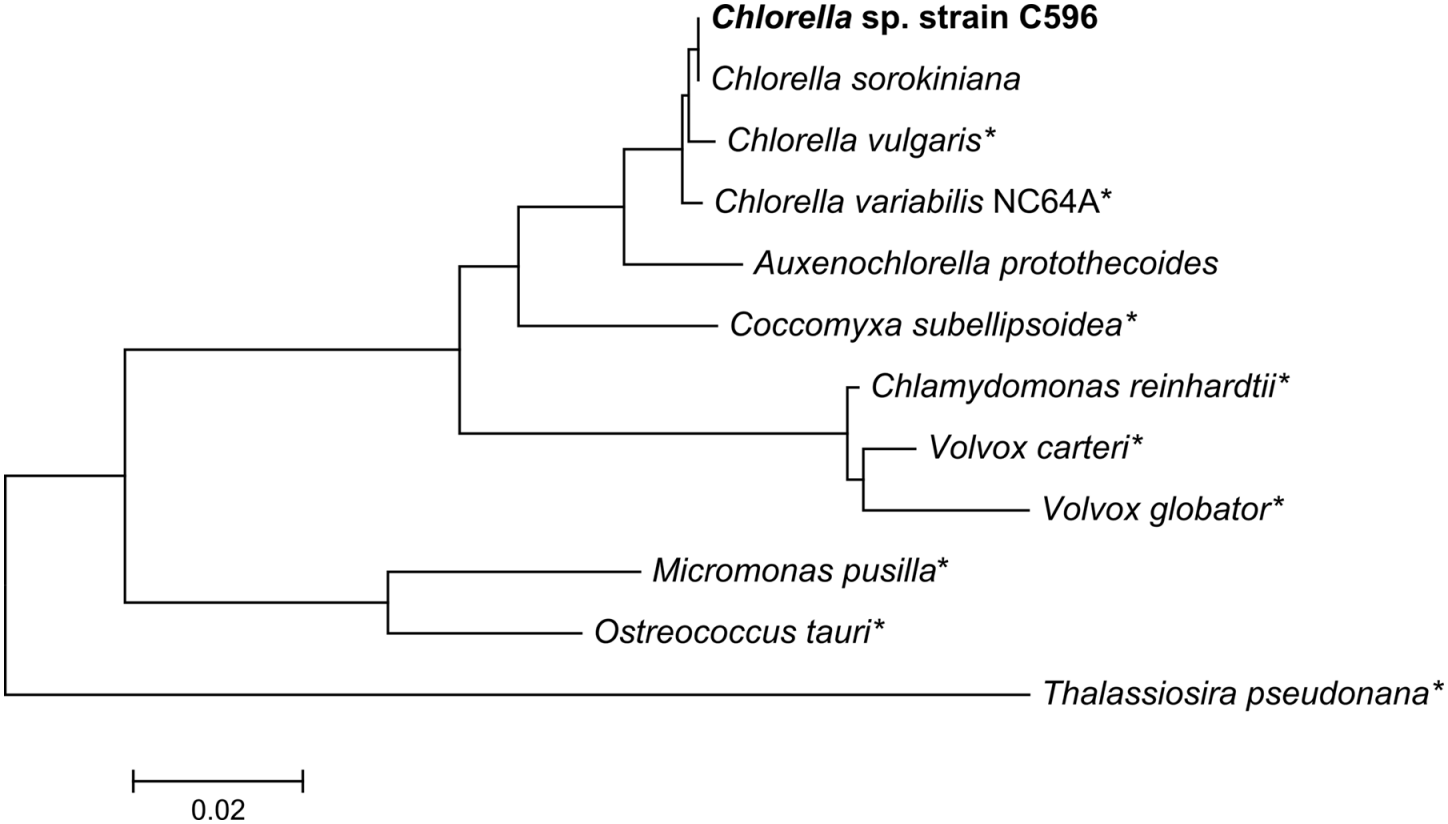
18S rRNA phylogenetic tree for marine and freshwater green algae and a marine diatom outlier. The asterisks indicates organisms with available genomes in the NCBI database. The representative full-length 18S rRNA sequences were aligned with MUSCLE in MEGA 6.

To confirm the phylogenetic assignment of this strain, the 18S rRNA sequence of *Chlorella* strain C596 was sequenced and aligned with the 18S rRNA sequences of other representative freshwater and marine strains in the Chlorophyta division and with one model marine diatom, *Thalassiosira pseudonana*. Using sequence similarities, a phylogenetic tree was constructed (Fig 1). *Chlorella* strain C596 is closely related to other *Chlorella* strains within the division Chlorophyta, in the class Trebouxiophyceae (as previously defined; [30, 52]). The most closely related strain is *C. sorokiniana* (98% 18S rRNA sequence identity), a free-living marine strain and a good lipid producer [53]. Also based on its position within this phylogenic tree, our strain appears to be closely related to the five other members of the *Chlorella* genus identified as good lipid accumulators in a recent survey of 37 lipid-rich microalgae [54].

### Growth Characteristics

**Fig 2:**
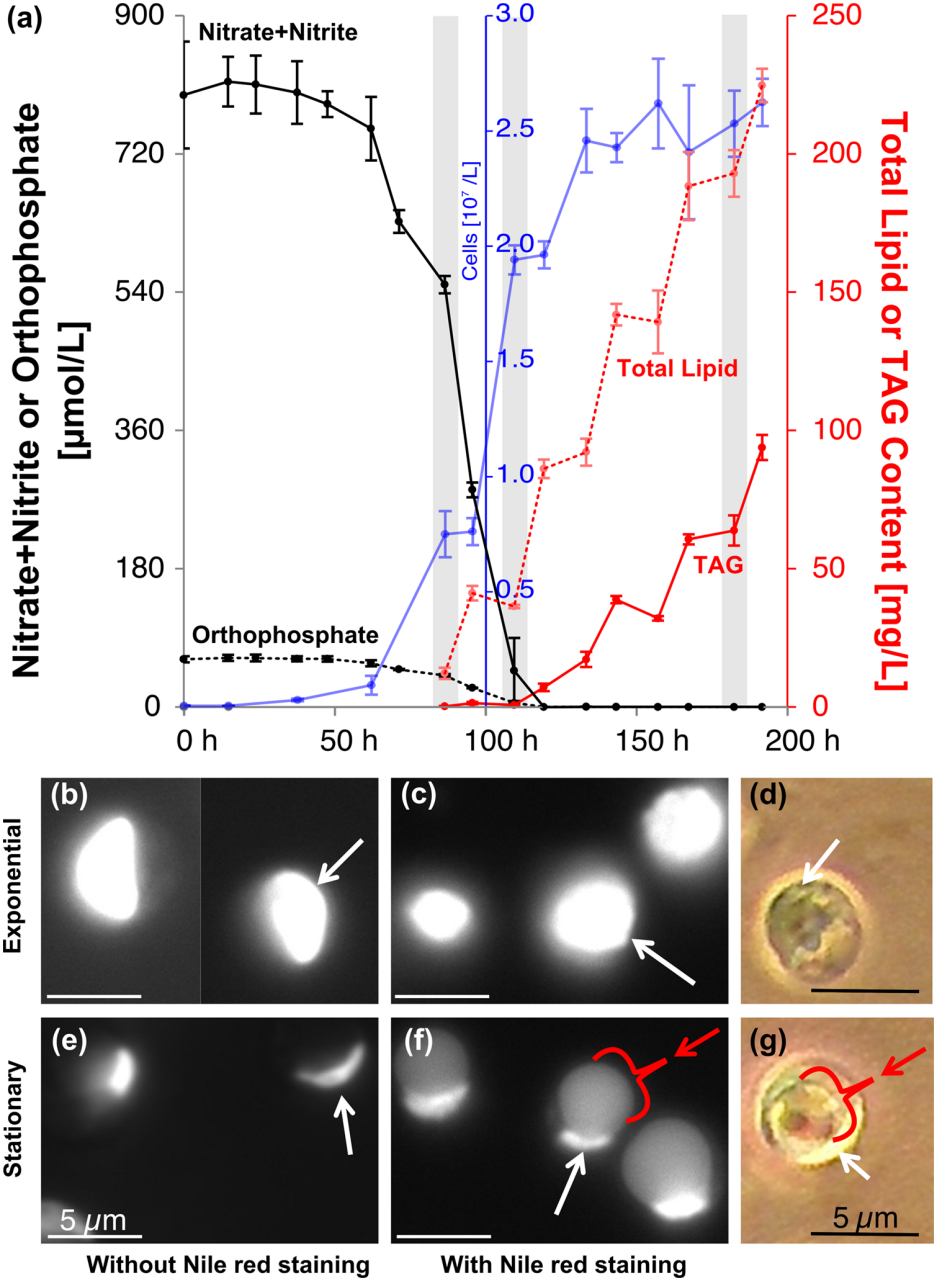
Lipid accumulation in *Chlorella* strain C596. (a) Time-course of a batch experiment culminating in N and P co-limitation (N:P = 13:1): nitrate+nitrite (black, solid), phosphate (black, dashed), cells (blue), TAG (red, solid), and total lipids (red, dashed) concentrations; error bars represent the 95% confidence interval. (b-g) Fluorescence and phase contrast microscopy of strain C596 cells grown in batch culture culminating in P limitation (N:P = 30:1). Samples were withdrawn during the exponential phase (b-d) and four days after onset of stationary (e-g) growth phase. The fluorescence microscopy reveals the chloroplast (autoflourescence, bright white) and Nile red stained lipids (in c and f; grey). The red arrows and brackets indicate intercellular TAG locations. The white arrows highlight the chloroplast.

Under optimized growth conditions (temperature and light with CO_2_ supplementation), strain C596 averaged a specific growth rate of 1.64 d^−1^ during the nutrient-replete exponential growth phase, began to accumulate TAGs as the external reserves of nitrate (then phosphate) were exhausted, and then entered the stationary growth phase (Fig 2a). In the current study, the culture bioaccumulated 225 ± 3 mg L^−1^ (46.5 ± 1.8 mg L^−1^ d^−1^) of total lipids and 94 ± 2 mg L^−1^ (26.2 ± 0.4 mg L^−1^ d^−1^) of TAGs within 72 h of nutrient depletion. Estimates of total lipid and TAG content relative to dry weight in C596 for this experiment are 66% and 28%, respectively, assuming an average cellular radius of 2.5 μm (Fig 2b–2g) and a dry weight fraction of 20%. Previously, the maximum lipid accumulation rate in this strain was reported to be as high as 61 mg L^−1^ d^−1^ (in cells that were approximately 45% TAG by weight) [55]. These rates approach those obtained with *Nannochloropsis* sp. grown under optimized conditions (75–300 mg L^−1^ d^−1^; [56]) and are at the high end of the range of lipid and TAG levels relative to dry weight reported for algae generally and Chlorophytyes specifically (5–50% lipid by dry weight; [6]).

The accumulation of lipids is also apparent in the microscopy images of C596 cells from a batch culture grown with excess nitrogen (N:P = 30:1; Fig 2b–2g). During the exponential growth phase, chlorophyll auto-fluorescence is observable as intense white portions of the cell (Fig 2b and 2c) which diminish in size and intensity on reaching the phosphorous-induced stationary phase of growth (Fig 2e and 2f). Nile red was used to stain the internal lipids, corresponding to the lighter gray droplet of TAG readily apparent in the cells during stationary growth (Fig 2f), at which point the chloroplasts have been pushed to the edge of the cell by the TAG-rich lipid bodies. Chloroplast migration is also evident in the phase contrast images (Fig 2g). Similar observations of the chloroplast being pushed to the surface of the cell during lipid accumulation have been reported for *C. variabilis* NC64A cells subjected to nitrogen deprivation [16] and for *Nannochloropsis* [57].

### Heterotrophic Growth and Salt Response

**Fig 3:**
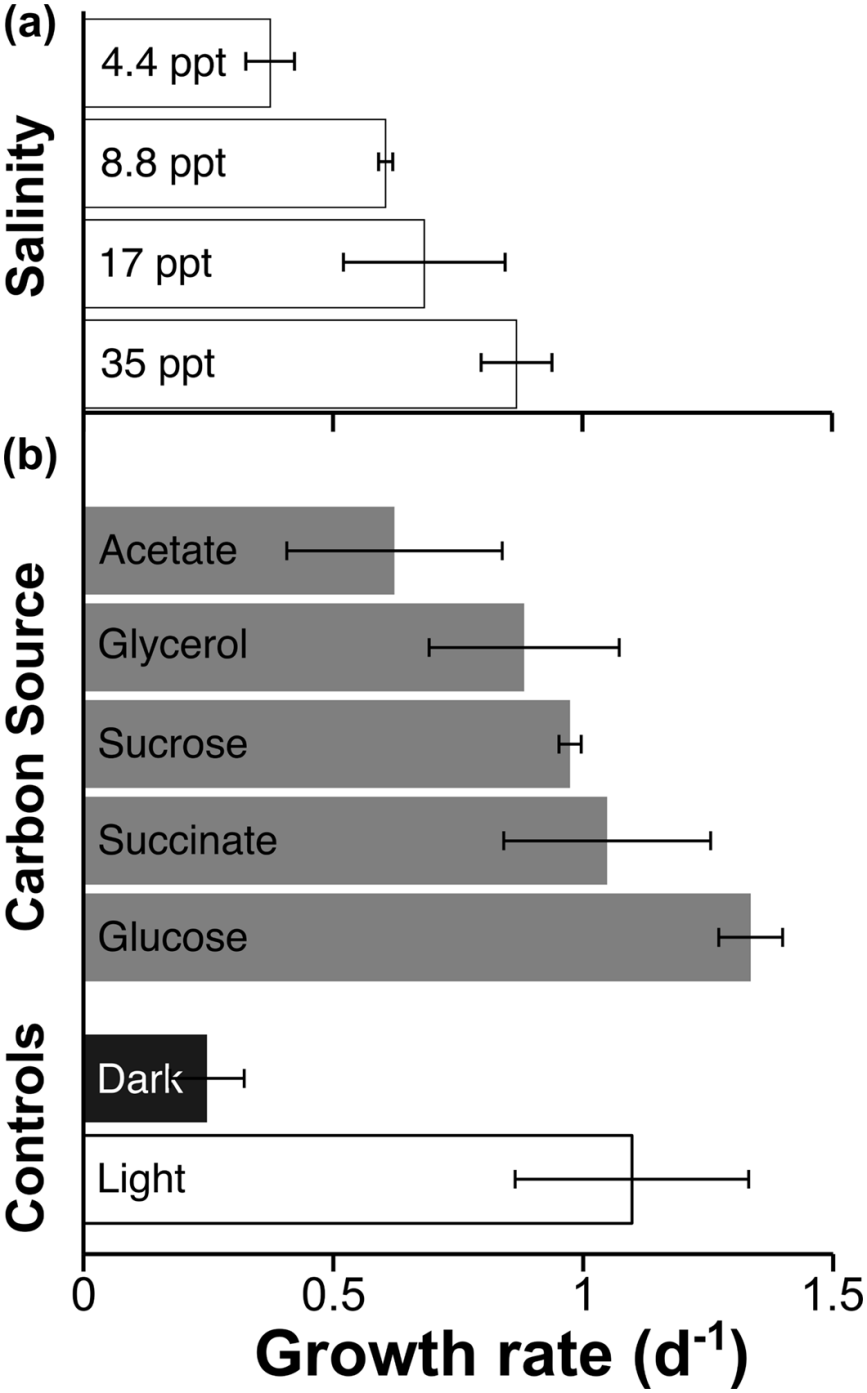
Specific growth rates of *Chlorella* strain C596. Growth rates (d^-1^, based on cell density over time) are shown for (a) different salinities and (b) various carbon substrates. White bars indicate photosynthetic growth with a PPFD of 80 μmol photons m^−2^ s^−1^; gray bars indicate grown in the dark plus a carbon source at a final concentration of 20 mmol L^−1^; the black bar indicates growth in the dark. Values are the means of triplicate biological replicates (n = 3); error bars represent ±1 standard error (SE).

*Chlorella* strain C596 can tolerate a wide range of salinities, but grew slower at reduced salinities (Fig 3a). The specific growth rates achieved by C596 in the reduced-salt Aquil media were maintained for three subsequent generations (data not shown). The highest growth rate of this *Chlorella* species was obtained at a salinity level of 35 ppt, the salinity of our synthetic Aquil media [34]. This salinity value is higher than the optimal value of 20 ppt reported for *A. protothecoides* [58]. However, higher salinities (up to 60 ppt) do not substantially reduce the intrinsic growth rate of strain C596 under nutrient replete conditions (Dr. Z. Johnson, personal communication, May 23, 2015).

In the dark, strain C596 was able to grow using 20 mmol L^−1^ glucose, sucrose, succinate, and glycerol at heterotophic growth rates that rivaled the photosynthetic growth rate of ∼1 d^−1^ (Fig 3b). Only acetate resulted in statistically lower growth rates of 0.6 d^−1^ ± 0.2 (47% less than phototrophic rates; p-value < 0.03, Student’s t-test). Additionally, glucose supported the fastest heterotrophic growth rate compared to the other carbon substrates (36% greater with glucose than succinate, p-value < 0.003, Student’s t-test), and was 23% greater than photoautotrophic growth at the identical temperature but with lower than optimal PPFDs (p-value < 0.1, Student’s t-test).

Both *C. sorokiniana* and *C. vulgaris* have been shown to grow heterotrophically on glucose as the sole carbon and energy source, with 29% and 26% improved growth rates for *C. sorokiniana* and *C. vulgaris*, respectively, for cultures grown with 27 mmol L^−1^ glucose compared to purely photosynthetic growth [59, 60]. The photoautotrophic growth rates of strain C596 (∼1.2 - 1.6 d^−1^) reported in the present study are at the high end of previously reported growth rates for other *Chlorella* species (e.g., *C. sorokiniana*, 1.02 - 1.6 d^−1^) [61]. Though we did not investigate the effect of heterotrophic growth on lipid accumulation in C596, *C. vulgaris* was noted to have a higher cellular lipid content under autotrophic growth whereas higher lipid productivity (on a mass per day basis) under heterotrophic growth [62]. Our results reveal strain C596 to be a versatile mixotrophic algae strain capable of utilizing a broad range of organic substrates over a wide range of salt concentrations—desirable characteristics in an alga for industrial biofuel applications.

### Transcriptome Annotation

To link the accumulation of lipids to the expression of certain transcripts, we sequenced the transcriptome over a time course when nitrogen was exhausted and TAG accumulation occurred (Fig 2). Our base library was constructed from 11.4 million read pairs (5.7 Gbp of sequence), and the assembled transcriptome resulted in 131,470 transcripts. Of these assembled transcripts, 13,036 were treated as detected transcripts (at least five transcripts in at least one sample; S2 File). The sequences in the detected transcriptome were searched against NCBI’s nr database using the BLASTx algorithm. All but 254 of these sequences displayed at least one hit to the nr database with an e-value cutoff of 10^−5^. In total, 137,484 significant hits were returned, of which *C. variabilis* (19,027 hits), *Coccomyxa subellipsoida* (15,828 hits), *Volvox cartei* (9,772 hits), and *C. reinhardtii* (9,283 hits) were the top four algae in rank order of the number of hits and combined comprised 39.2% of the total significant hits (*A. protothecoides* was not available in this database). In total, 8,903 transcripts in our library were identified as displaying homology to a Chlorophyta species. These 8,903 were considered as the expressed transcriptome in this study.

To estimate what fraction of the full transcriptome was detected as expressed transcripts in this study, we compared the expressed transcriptome to three other fully sequenced species in a tBLASTn analysis: *C. variabilis* (which returned the most hits in the BLASTx analysis above), *Chlorella* UTEX1228 (the complete list of predicted proteins is available at greenhouse.lanl.gov), and *A. protothecoides* (a closely related genus [22]). In this analysis, the expressed transcriptome displayed sequence homology to 79% (7,705 out of 9,791 predicted transcripts with an average hit length of 213 amino acids (aas)), 64% (7,760 out of 12,169 predicted transcripts with an average hit length of 302 aas), and 77% (5,752 out of 7,431 predicted transcripts; with an average hit length of 222 aas) of all of the transcripts predicted for *C. variabilis*, *Chlorella* UTEX1228, and *A. protothecoides*, respectively. These results suggest that our transcript library captured a large and representative fraction of genome-encode transcripts in *Chlorella* strain C596. We estimate that the size of the C596 genome is approximately 30 Mbp (unpublished), between that of *A. protothecoides* (22.9 Mbp; [22]) and *C. variabilis* NC64A (46.2 Mbp) or *C. pyrenoidosa* FACHB-9 (56.8 Mbp) [21].

### Differentially Regulated Transcripts between Nutrient-Replete and Nutrient-Limited Cultures

The transcriptional regulation of biochemical pathways in response to changes in environmental stimuli was monitored using the RNA-seq data; in particular we sought to ascertain which transcripts were significantly regulated during the transition to nutrient-limited growth and TAG accumulation. A global edgeR analysis was performed on the time course samples which included biological duplicates from three time-points and comprised 4.1 million read pairs (2.1 Gbp of sequence). Applying all of the filters described in the Materials and Methods, RNA-seq data from these samples yields a subset of the expressed transcriptome (6,554 of the 8,903 transcripts). When the 86 h sample (external concentrations of nitrate and phosphate still considered saturating for growth, Fig 2) was compared to the 109 h sample (the culture during the transition phase), 397 of these transcripts were differentially expressed (greater than two fold-ratio change and a p-value < 0.05). When comparing the transcriptome of the organism during the transition phase (109 h samples) to the nutrient-depleted (182 h) samples, the edgeR analysis identified 2,023 transcripts as significantly differentially expressed, of which 1,099 were up-regulated and 924 were down-regulated (S3 File). A more pronounced shift in gene expression was thus revealed for the 182/109 h comparison relative to the 109/86 h comparison (3.3 times more genes were differentially expressed).

The substantial RNA turnover in later cultures (182 h) is supported by the Bioanalyzer traces of the extracted total RNA. We note that when *Chlorella* strain C596 transitioned from the exponential growth phase (86 and 109 h) to the stationary phase (182 h; S2 Fig), the RNA pool displays dampened dominant rRNA peaks. However, this degradation is not noted in late stationary phase samples, suggesting that the degradation of RNA in the early stationary phase is representative of a high amount of RNA turnover in preparation for stationary phase and not an artifact of the extraction process. The 182/109 h comparison was explored further to identify transcripts that significantly respond to nutrient limitation and TAG accumulation.

**Fig 4:**
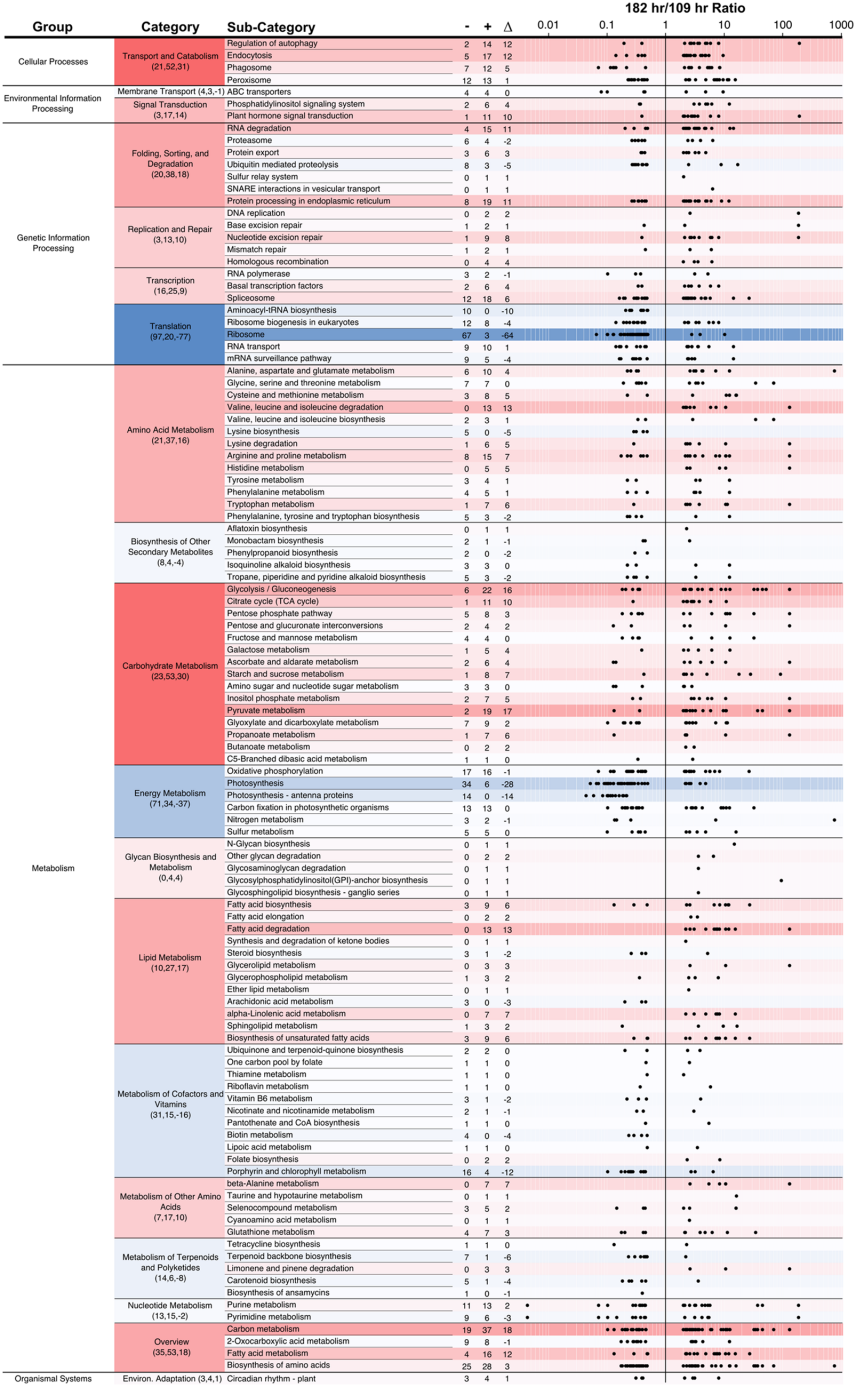
Differentially expressed genes between the 182 h versus 109 h samples broken into KEGG categories. Genes are considered differentially expressed when the edgeR p-value < 0.05 and the fold-ratio > 2 or < 0.5. The assignment of a transcript to a category is based upon the KEGG annotation of orthologs in *C. variabilis* NC64A. The axis of the dot plot displays the ratio of 182/109 h on a log scale. The numbers in the category column represent the number of genes down-regulated (-), up-regulated (+), and the difference between these (Δ). The color shading is based on the Δ (positive, red; negative, blue).

The sequences for the transcripts that were significantly regulated between the 182 h and 109 h time points were compared against the KEGG annotations for *C. variabilis* in a tBLASTn search. In total, 475 of the 2,023 differentially expressed transcripts were assigned KEGG categories in this analysis (the unmapped transcripts had no associated KEGG category or did not map to *C. variabilis*). The KEGG-assigned transcripts were binned into five top-level Groups in the KEGG hierarchy (Organismal Systems, Cellular Processes, Environmental Information Processing, Genetic Information Processing, and Metabolism) which were sub-divided into 17 Categories, and 111 Sub-categories (out of a possible 118 identified for *C. variabilis*). The numbers presented in Fig 4 are the number of unique differentially expressed transcripts matching a KEGG organizational level. Because of the potential multiple assignment of KEGG sub-categories per transcript (the 475 transcripts were assigned an average of 2.5 categories per transcript because enzymes map to multiple pathways), the values for the total number of unique transcripts up- or down-regulated in the category level are not the simple sum of the sub-category level.

A number of notable metabolic shifts are reflected in the gene expression profiles after strain C596 entered into nutrient limitation (Fig 4). Within the Metabolism KEGG Group, the majority of genes associated with Carbohydrate Metabolism (23 down, 53 up) and Lipid Metabolism (10 down, 27 up) categories were up-regulated. Additionally, the majority of the transcripts identified in the photosynthesis sub-category (34 down, 6 up) and the photosynthesis—antenna proteins sub-category (14 down, 0 up) within the Energy Metabolism Category are down-regulated. Taken together, these results suggest that strain C596 is scaling back the photosynthetic apparatus when substantially enhancing lipid and carbohydrate metabolism. A similar pattern was seen in a closely related species, *A. protothecoides*, which also displayed up-regulation of carbon metabolism-related transcripts and down-regulation of photosynthesis related transcripts on entering the TAG accumulation phase under heterotrophic growth when compared to autotrophic growth [22].

The TAG accumulation phase in algae is known to be decoupled from organismal growth (Fig 2; [63]). The differential response of various categories within the Genetic Information Processing Group highlights this decoupling (Fig 4). Within the Translation Category (97 down, 20 up), ribosome associated transcripts are significantly down-regulated, suggesting that fewer ribosomes are being produced and matured. Additionally, within the Folding, Sorting, and Degradation Category, transcripts related to RNA degradation are up-regulated (4 down, 15 up), corroborating a higher RNA turnover in the 182 h sample compared to the 109 h sample (S2 Fig).

### TAG Synthesis Pathway Regulation

**Fig 5:**
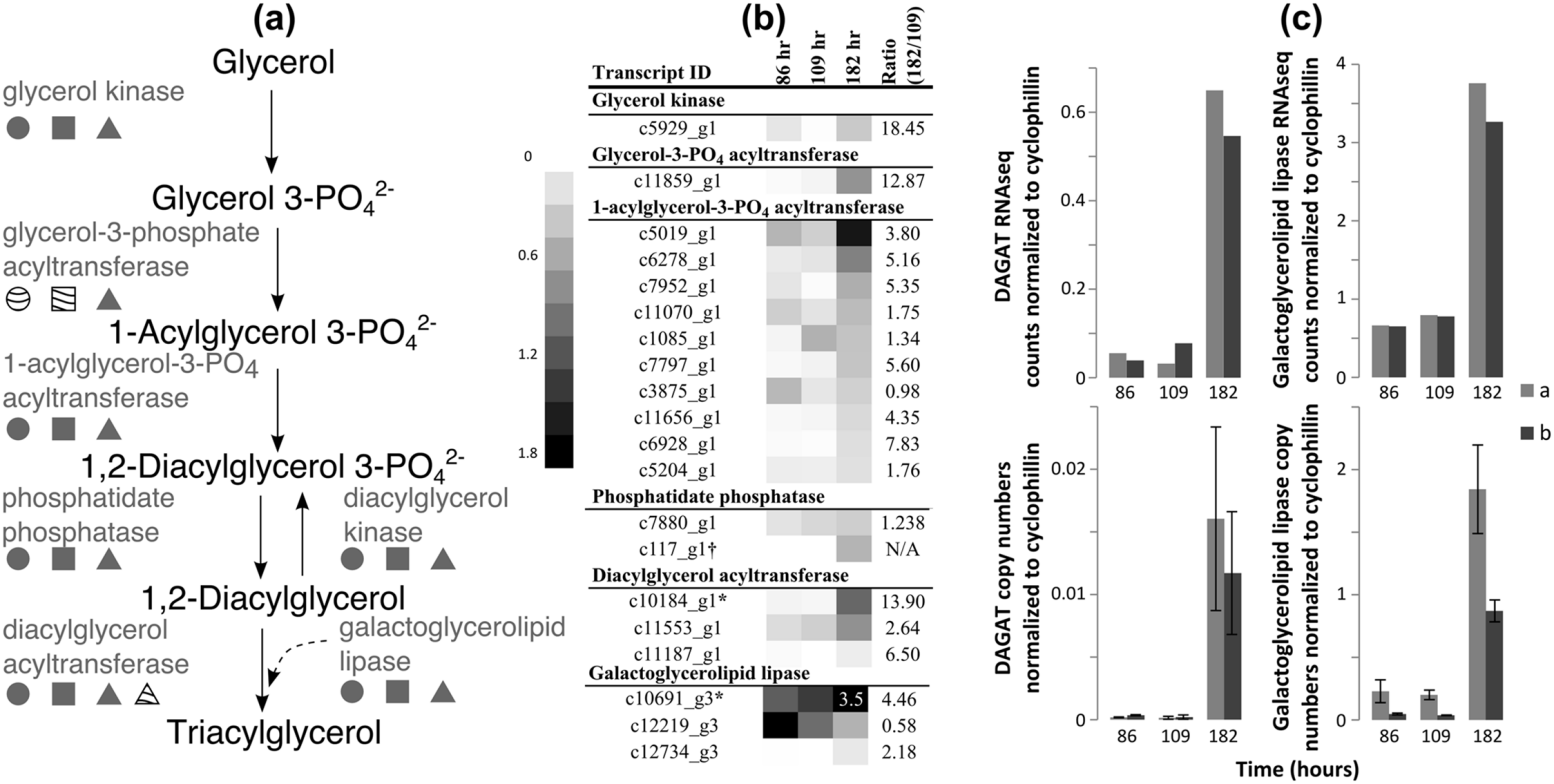
Detection and monitoring of the TAG biosynthesis pathway in *Chlorella* strain C596. (a) The final steps of the TAG biosynthesis pathway. The symbols indicate whether one or more transcripts encode the enzyme at that step is present for *Chlorella* strain C596 (circle), *C. variabilis* NC64A (square), or *C. reinhardtii* (triangle). Grey shading indicates presence. Lined shading for the glycerol-3-phosphate acyltransferase indicates a bacterial-type homolog. Lined shading for the diacylglycerol acyltransferase indicates a secondary homolog in *C. reinhardtii*. (b) Heatmaps of the cyclophillin-normalized values for the putative transcripts for the TAG accumulation pathway and galactoglycerolipid lipase in samples taken during exponential growth (89 and 109 h) and during lipid accumulation (182 h). The intensity of the shading represents cyclophillin normalized values ranging from 0 to 1.8; note, one transcript, c10691_g3, falls outside of this range and the appropriate box is labeled. The * symbol represents those transcripts for which RT-qPCR primers were designed. The † symbol represents the transcript whose ratio could not be calculated because the transcript was not detected in the 109 h sample. (c) Comparison of the RNA-seq (top row) and RT-qPCR results also normalized to cyclophillin (bottom row) for the predicted DAGAT (left column) and galactoglycerolipid lipase (right column) for two biological duplicates (a and b). The error bars indicate the 95% confidence interval of the technical replicates run for the RT-qPCR.

Our KEGG based analysis only focused on genes that were differentially regulated and were annotated with a KEGG descriptor based on homology with *C. variabilis* NC64A. Broadening the analysis to consider all transcripts detected in the strain C596 samples that displayed homology to a Chlorophyta protein in the NCBI nr database, we identified genes associated with the last steps of the TAG synthesis pathway (Fig 5a). In Fig 5a, the circle, square, and triangle represent the presence of sequences predicted to encode for enzymes that catalyze each of these steps in strain C596, *C. variabilis* NC64A, or *C. reinhardtii* [64], respectively. For the majority of the steps in Fig 5a, the sequences were present and displayed strong homology in all three strains. However, the *Chlorella* strains deviated from the *Chlamydomonas* in the sequence composition of the glycerol-3-phosphate acyltransferase; the *Chlorella* strains display a bacterial type homolog whereas *C. reinhardtii* displays a plant-like homolog.

The cyclophillin (a housekeeping gene) normalized time-course for the transcriptomic abundance of these TAG biosynthesis pathway homologs is displayed in Fig 5b. The results obtained using actin as the housekeeping gene were quantitatively similar (data not shown). As the organism enters the TAG accumulation phase, transcripts representing the final biosynthesis steps for TAG and the galactoglycerolipid lipases are up-regulated. Multiple potential paralogs are observed in C596 for several of these steps. This is also true in *C. reinhardtii* which was noted to have two 1-acylglycerol-3-phosphate acyltransferases, three phosphatidate phosphatases, six DAGATs and three lipases [65]. The transcripts encoding for the enzymes of the first two steps (c5929_g1, glycerol kinase; c11859_g1, glycerol-3-phosphate acetyltranseferase) displayed a high up-regulation (18 and 13 fold increases, respectively) (Fig 5b). Additionally, the transcripts encoding for two enzymes often explored in other studies, c10691_g3 (a galactoglycerolipid lipase paralog) and c10184_g1 (a diacylglycerol acyltransferase paralog; DAGAT), were also up-regulated (4.5 and 14 fold, respectively). DAGAT is responsible for the final step in TAG biosynthesis, and a substantial up-regulation was also noted for DAGAT (approximately a 140 fold-change) under nitrogen limitation in another study [16]. Additionally, the galactoglycerolipid lipase is predicted to be critical in the final formation of TAG from galactolipids in *C. reinhardtii* [64, 66], but has not been previously identified as playing a role in *Chlorella*. A similar pattern of up-regulation of lipid biosynthesis genes was noted in *C. sorokiniana* when the organism experienced higher carbon-dioxide partial pressures [27].

Primers for the most strongly up-regulated galactoglycerolipid lipase and DAGAT transcripts were designed, and a RT-qPCR analysis on the identical samples was run to confirm the results of the RNA-seq analysis with respect to the response of the expression of these genes to nitrogen starvation. The RT-qPCR analysis confirmed that both the galactoglycerolipid lipase and DAGAT are up-regulated in each of the biological duplicates (Fig 5c). Both methods displayed a higher overall transcript abundance for the galactoglycerolipid lipase than for the DAGAT, but the cyclophillin normalized ratios of the galactoglycerolipid lipase and DAGAT between the 182 h to the 109 h time points determined via the RNA-seq analysis (4.5 ± 0.5 and 14 ± 13 fold higher, respectively) were 3.6 and 5.8 times lower than the ratios obtained using the RT-qPCR analysis (16 ± 14 and 81 ± 49 fold higher, respectively). Previous studies comparing RNA-seq ratios and RT-qPCR ratios in *Chlamydomonas* noted that the RNA-seq ratios were, on average, approximately four times lower than the reported RT-qPCR ratios [67].

The consistent up-regulation of these transcripts in both the RNA-seq analysis and the RT-qPCR analysis emphasizes their potential role in TAG accumulation. In green algae, DAGAT is predicted to be and enzymatically shown to be the terminal enzyme in TAG biosynthesis, transferring an additional acyl-glycerol onto a diacyl-glycerol backbone [68, 69]. By contrast, the lipase is predicted to be involved indirectly in the accumulation of TAG via the metabolism of chloroplast monogalactosyldiacylglycerol in green algae such as *C. reinhardtii* [66]. In previous studies that employed gene-knockout and over-expression techniques, *C. reinhardtii* was shown to require the expression of this lipase to accumulate TAG under nitrogen starvation conditions [64–66, 70]. This function contrasts with the role played by other lipases that recycle TAG and slow the accumulation of the lipid [71]. Our results are consistent with two distinct roles for individual lipases as well: one which is expressed under nutrient-replete conditions (c12219_g3) and the other which is expressed when the TAG accumulation begins during nutrient-depleted conditions (c10691_g3) (Fig 5). Confirmation of these roles would require manipulation of individual enzyme levels (e.g., by knock-out or over-expression) and a measurement of phenotypic effects similar to studies performed in *C. reinhardtii* [69, 71].

## Summary

This study investigated the growth versatility of *Chlorella* strain C596 and used transcriptome sequencing data to discover cellular responses of this marine microalga to nutrient-limitation. The organism was determined to bioaccumulate industrially relevant TAGs in response to nutrient limitation. The euryhaline nature of strain C596 was demonstrated by the ability of this alga to grow over a wide range of salinities, ranging from 4.4 to 35 ppt, although the cultures grew fastest at the higher salinities. Additionally, the heterotrophic growth rate achieved by this strain when grown in the dark rivaled that of the specific growth rate obtained using inorganic nutrients under photosynthetic light conditions, allowing for a potential industrial practice of supplementing photoautotrophic growth with carbon substrates to increase growth and possibly to enhance TAG productivity.

The transcript library assembled from strain C596 contained orthologs to more than 75% of genes in *C. variabilis* NC64A and *A. protothecoides*—suggesting that a large fraction of the strains’ transcripts were accounted for in the assembled composite transcriptome. Additionally, a putative TAG biosynthesis pathway was identified through a comparison with *C. variabilis* NC64A and *C. reinhardtii*. Overall, the transcripts encoding for the majority of enzymes involved in this pathway displayed a distinct up-regulation when entering the nutrient limited lipid accumulation phase. A RT-qPCR analysis confirmed the strong up-regulation of two key enzymes: DAGAT and a lipase involved in the recycling of galactolipids into TAGs.

## Supporting Information

S1 Fig Flowchart displaying the assembly and annotation pipeline. (TIFF)

S2 Fig RNA integrity during the growth phases for the time course samples. (TIFF)

S1 Table qPCR primers designed for selected target transcripts. “LA” designates Long Amplicons used to create quantitative standards for qPCR.
(PDF)

S1 File Chlorophyta 18S rRNA sequences used to generate the phylogenetic relationships. (FASTA)

S2 File Assembled contig FASTA library considered the detected transcriptome. (FASTA)

S3 File RSEM expected counts and TPM values per Trinity assembled contig for the time-course co-limitation experiment. (XLSX)
