# Supplementary figures and images for: Use of De Novo Transcriptome Libraries to Characterize a Novel Oleaginous Marine Chlorella Species during the Accumulation of Triacylglycerols
Source: PLoS One. 2016 Feb 3;11(2):e0147527. doi: 10.1371/journal.pone.0147527 (PMC4740408; doi:10.1371/journal.pone.0147527)

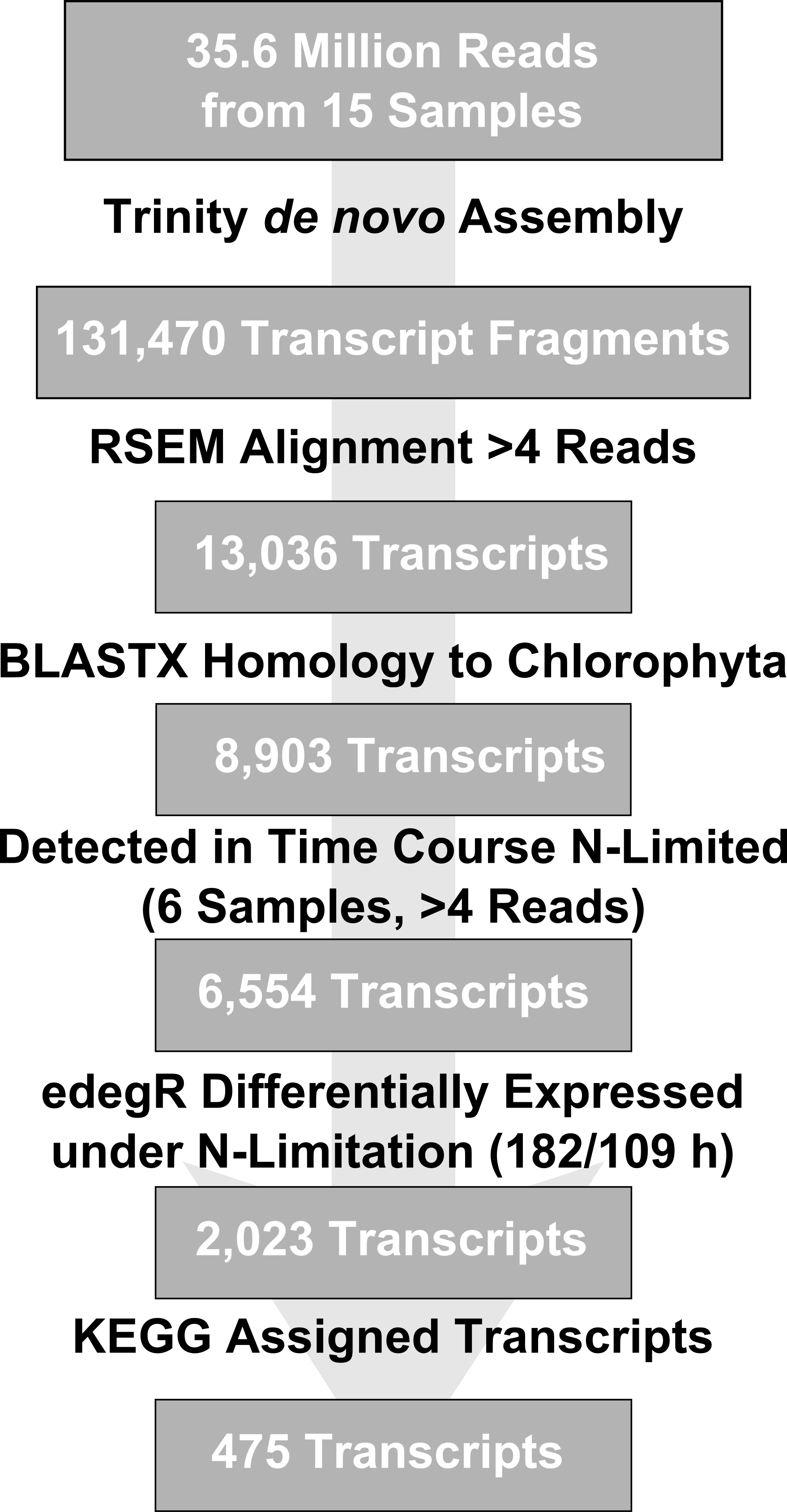

Supplement: S1 Fig — (TIFF) [file pone.0147527.s001.tiff]

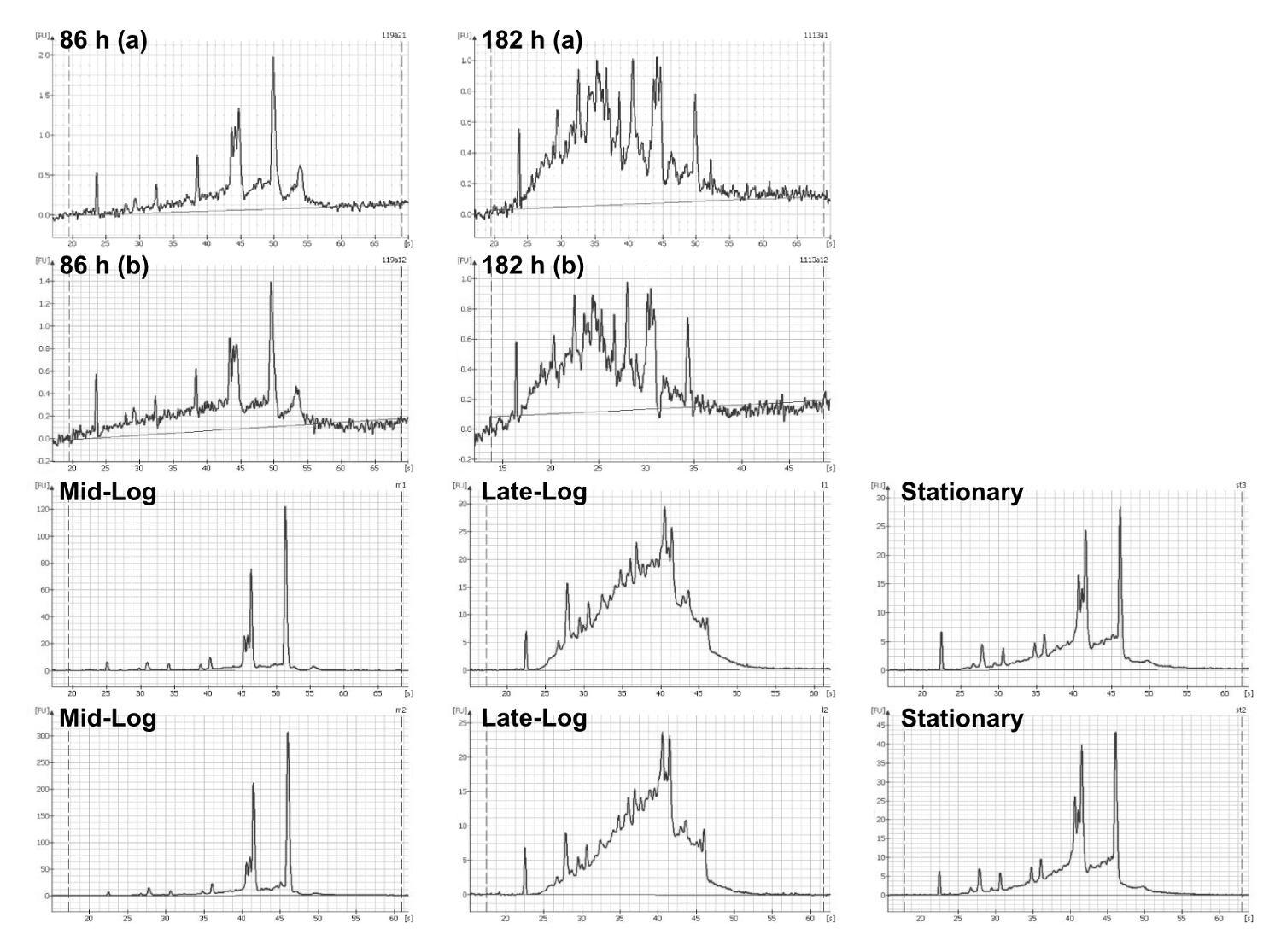

Supplement: S2 Fig — (TIFF) [file pone.0147527.s002.tiff]
