# Supplementary material for: Use of De Novo Transcriptome Libraries to Characterize a Novel Oleaginous Marine Chlorella Species during the Accumulation of Triacylglycerols
Source: PLoS One. 2016 Feb 3;11(2):e0147527. doi: 10.1371/journal.pone.0147527 (PMC4740408; doi:10.1371/journal.pone.0147527)
Supplement: S1 Table — “LA” designates Long Amplicons used to create quantitative standards for qPCR. (PDF) [file pone.0147527.s003.pdf]

Target	Forward Primer (5'-3')	Reverse Primer (5'-3')	Annealing Temp	Amplicon Length (bp)
DAGAT	AAAACACGATGATGCCTGCC	GGAGTTCTACAAGGAGTGGTGG	56°C	152
DAGAT LA	AAAGCAGAAGGACAGCCAGAA	TTGTGGAGAACAGCCTTGGG	56°C	494
TAG Lipase	ACATTTGACCCGGTGGTGTT	ACCACAGCAGCCTCATACAC	60°C	77
TAG Lipase LA	AGACGATGACCAGACGCAC	CCCCTCCGCAAAAACATGAG	60°C	285
Cyclophillin	AAGGGTTTGGGAAAGAGCGG	ACCAGTGCCATTACCAGCAG	60°C	155
Cyclophillin LA	GGGCAGAATAGAGATGACGCT	CACAGGAAGAACTGGGAGCC	60°C	315
16S rRNA	AGAGTTTGATCCTGGCTCAG	GGCTACCTTGTTACGACTT	51°C	1500
18srRNA	AACCTGGTTGATCCTGCCAGT	GATCCTTCTGCAGGTTCACCTAC	57°C	1800
